# Landscape and Predictive Significance of the Structural Classification of *EGFR* Mutations in Chinese NSCLCs: A Real-World Study

**DOI:** 10.3390/jcm12010236

**Published:** 2022-12-28

**Authors:** Linping Gu, Huayan Huang, Zhangwendi Xu, Xiaomin Niu, Ziming Li, Liliang Xia, Yongfeng Yu, Shun Lu

**Affiliations:** Department of Shanghai Lung Cancer Center, Shanghai Chest Hospital, School of Medicine, Shanghai Jiao Tong University, 241 Huaihai West Road, Shanghai 200030, China

**Keywords:** EGFR TKIs, time to treatment failure, EGFR mutations, structural classification, NSCLC

## Abstract

**Background:** Non-classical *EGFR* mutations demonstrate heterogeneous and attenuated responsiveness to EGFR TKIs. Non-small cell lung cancer (NSCLC) patients with atypical *EGFR* mutations have limited therapeutic options. A recent study established a novel structural-based classification of *EGFR* mutations and showed its value in predicting the response to TKI. We sought to interrogate the distribution of different structural types and to validate the predictive value in Chinese NSCLCs. **Methods:** A total of 837 tumor samples were retrospectively recruited from 522 patients with unresectable *EGFR*-mutant NSCLC. *EGFR* mutations were classified into four groups: classical-like, T790M-like, Ex20ins-L, and PACC. Treatment information and clinical outcomes were obtained from 436 patients. The time to treatment failure (TTF) was determined on a per-sample basis. **Results**: Of the 837 *EGFR*-mutant samples, 67.9%, 18.5%, 9.0%, and 3.1% harbored classical-like, T790M-like, PACC, and Ex20ins-L mutations, respectively. Thirteen (1.6%) samples carried mutations beyond the four types. Among the 204 samples with atypical mutations, 33.8%, 36.7%, 12.7%, and 10.3% were classical-like, PACC, Ex20ins-L, and T790M-like, respectively. In patients with PACC mutations, second-generation TKIs demonstrated a significantly longer TTF than first-generation TKIs (first-line: 15.3 vs. 6.2 months, *p* = 0.009; all-line: 14.7 vs. 7.1 months, *p* = 0.003), and a trend of longer TTF than third-generation TKIs (all-line: 14.7 vs. 5.1 months, *p* = 0.135). **Conclusions:** Our study depicted the landscape of structural types of *EGFR* mutations in Chinese NSCLC patients. Our results also suggest that the structural classification can serve as a predictive marker for the efficacy of various EGFR TKIs, which would guide therapeutic decision making.

## 1. Introduction

It has been reported that 10–15% of Caucasian patients and 30–35% of Asian patients with non-small cell lung cancer (NSCLC) harbor activating *EGFR* mutations [1]. Deletions in exon 19 (Ex19del) and L858R comprise 40–50% and 30–40% of all *EGFR* mutations, respectively. The use of first and second-generation EGFR tyrosine kinase inhibitors (TKIs) has substantially improved the clinical outcomes of NSCLC patients with the classical sensitizing mutation (L858R or Ex19del) [2,3,4]. The third generation TKI osimertinib, which was developed to selectively inhibit EGFR with a sensitizing mutation and resistant T790M, demonstrates remarkable efficacy as a first-line treatment, with a median progression-free survival (mPFS) of 18.9 months and median overall survival (mOS) of 38.6 months [5].

Mutations beyond L858R and Ex19del constitute 10–15% of the *EGFR* mutations found in NSCLC [1,6]. Among these atypical *EGFR* mutations, the most prevalent are exon 20 insertions, G719X, S768I, and L861Q. Up to 25% of uncommon mutations occurred concomitantly with other EGFR mutations, known as “compound mutations” [7]. The clinical data remain limited for these atypical *EGFR* mutations as patients with such mutations have generally been excluded from randomized trials. Based on the limited clinical evidence, atypical *EGFR* mutations demonstrate attenuated and heterogeneous responsiveness to EGFR TKIs. A combined analysis of LUX-Lung 2, LUX-Lung3, and LUX-Lung 6 showed that the response rate was 78%, 56%, and 100% for patients with G719X, L861Q, and S768I, respectively, and the mPFS was 13.8, 8.2, and 14.7 months, respectively [8]. Gefitinib has shown moderate sensitivity in patients harboring G719X or L861Q, with a response rate of 20% [9]. In a phase 2 study, osimertinib demonstrated an ORR of 53%, 78%, and 38% and mPFS of 8.2, 15.2, and 12.3 months in patients with G719X, L861Q, and S768I, respectively [10]. On the other hand, the limited efficacy of these EGFR-TKIs was observed in patients harboring exon 20 insertions, which stimulated the development of novel drugs specifically targeting 20 insertions [6]. Therapeutic options for other atypical *EGFR* mutations are scarcer as the corresponding clinical evidence is largely lacking.

A recent study established a structural classification of *EGFR* mutations on the basis of drug sensitivity data from cell lines. This novel classification categorized *EGFR* mutations into four groups: those distant from the ATP-binding pocket were termed as “classical-like”, “T790M-like” referred to mutations in the hydrophobic core, insertions in the loop at the C-terminal end of the αC helix in exon 20 were termed “Ex20ins-L”, and mutations predicted to be P-loop and αC-helix compressing were designated as “PACC”. This structure-based classification outperformed exon-based classification on stratifying mutations based on drug sensitivity and showed potential value for predicting EGFR-TKI efficacy in patients with NSCLC [11]. It is clinically crucial to validate this predictive role in Asian cohorts, given the high *EGFR* mutation prevalence seen in this population.

The present study aims to interrogate the distribution of different *EGFR* mutation types according to the structural classification and to validate its predictive role in a Chinese real-world cohort of NSCLC.

## 2. Materials and Methods

### 2.1. Patients and Study Design

**Figure 1:**
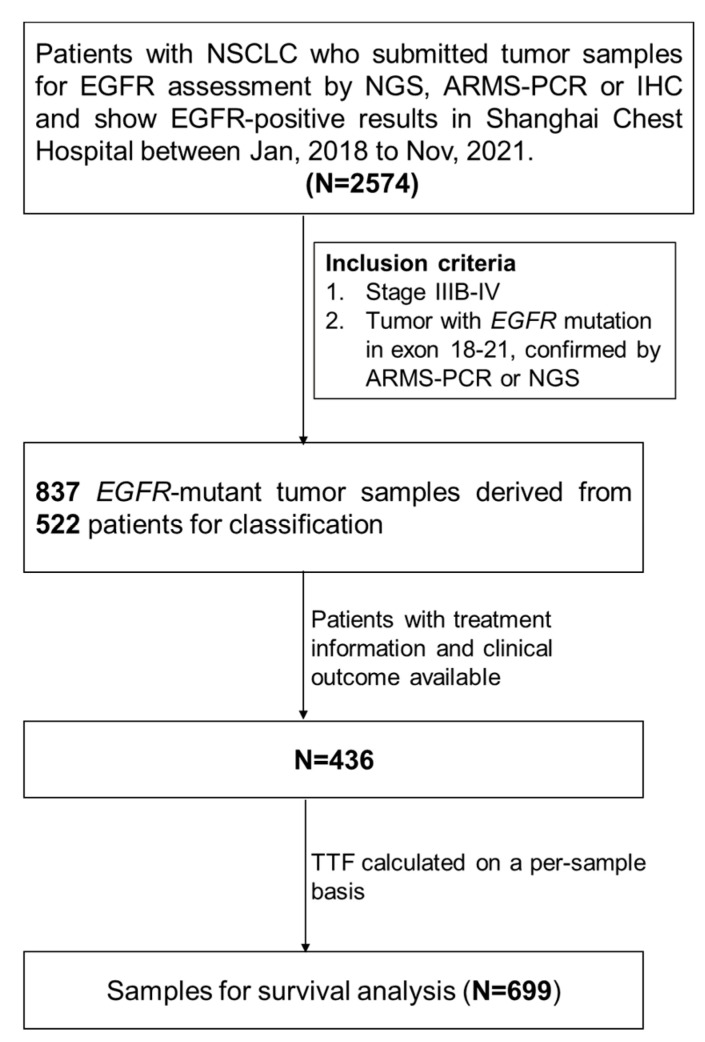
Flowchart of the study design.

Patients with NSCLC were retrospectively enrolled who submitted tumor samples for EGFR assessment in Shanghai Chest Hospital between January 2018 to November 2021. The inclusion criteria included: (1) pathologically confirmed unresectable NSCLC, stage IIIB-IV, and (2) a tumor harboring at least one *EGFR* mutation in exon 18–21, confirmed by amplification-refractory mutation system (ARMS) PCR or next-generation sequencing (NGS). The NGS and ARMS results were retrospectively retrieved. A total of 837 *EGFR*-mutant tumor samples from 522 patients were eventually included for structural classification (Figure 1), including 249 detected with ARMS-PCR and 588 with NGS. Patients’ demographic and clinical information was obtained from the medical records. Treatment information and corresponding clinical outcomes were available for 436 patients. The time to treatment failure (TTF) was determined on a per-sample basis and used for survival analyses. All of the procedures performed in studies involving human participants were in accordance with the 1964 Declaration of Helsinki and its later amendments. The study was approved by the Institutional Ethics Committee at Shanghai Chest Hospital (no. IS2123). Patient’s informed consent was waived due to the retrospective nature of the study.

### 2.2. ARMS-PCR

ARMS-PCR was performed with DNA extracted from formalin-fixed, paraffin-embedded (FFPE) samples using the *EGFR* Mutation Detection Kit (AmoyDx Diagnostics Co., Ltd., Xiamen, China) following the manufacturer’s instructions. The kit enables the detection of a total of 51 *EGFR* mutations in exon 18, exon 19, exon 20, and exon 21 (Table S1).

### 2.3. NGS

DNA was isolated from FFPE or biopsied tumor tissues using the QIAamp DNA FFPE tissue kit (Qiagen, Hilden, Germany), according to the manufacturer’s instructions. The NGS library was constructed, and target capture was performed using a panel consisting of 68 lung cancer-related genes (Lung Core, Burning Rock Biotech, Guangzhou, China). The panel covers whole exons 2, 3, 7, 8, 12, 15, 17–21, and 25. Prepared libraries were sequenced on MiseqDx or Nextseq500 (Illumina, Inc., San Diego, CA, USA) with paired-end reads and a target depth of 1000×. Variant calling and interpretation were carried out using an optimized bioinformatics pipeline that enables the accurate detection of somatic variants by discriminating sequencing artifacts from real mutations based on the methods described previously [12].

### 2.4. Classification of EGFR Mutations

Classical EGFR mutations in this study were defined as Ex19del/L858R with or without T790M. The remaining EGFR mutations were considered atypical mutations.

*EGFR* mutations were classified into four structural categories following the decision-tree criteria adapted from Robichaux et al. [11]: (1) Any mutations with T790M were assigned to the T790M-like group. (2) The remaining ones that had G719, L718, K757R, L718Q, S768I, D761Y, G724S, or E709_T710delinsD were assigned to the PACC group. (3) The remaining ones that included Ex20ins were assigned to the 20ins-L group. (4) The remaining ones that had L858R/833, Ex19del, or L861Q belonged to the classical-like group. Classification was subsequently adjusted manually to accord with the list of Robichaux et al. Table S2 shows mutations by structural type.

### 2.5. Statistical Analyses

R software (version 4.0.3) was used for all of the statistical analyses. Patient characteristics were summarized with descriptive statistics. Kaplan–Meier analysis was used to estimate the survival, and a log-rank test was used to determine the differences in the multiple survival metrics between groups. Statistical significance was defined as *p* < 0.05.

## 3. Results

### 3.1. Patients’ Characteristics

A total of 436 out of 522 patients had clinical outcomes and were included in the survival analyses. Patients’ clinical characteristics are summarized in Table 1. The median age of the cohort was 61 years old. Most patients were female (n = 234, 53.7%), had no history of smoking (n = 343, 78.7%), and were diagnosed with adenocarcinoma (n = 414, 94.9%). Four hundred twenty-one (96.6%) patients had stage IV disease. Three hundred and two patients received treatment with first generation EGFR TKI mainly as the first line (90.4%). One hundred and twenty-four patients received second generation EGFR TKI as first line (43.5%) or later-line (56.5%) treatment. Third generation EGFR TKI was administrated in 241 patients, predominately as later-line treatment (76.8%).

### 3.2. Classical and Atypical EGFR Mutations in the Chinese Cohort

**Figure 2:**
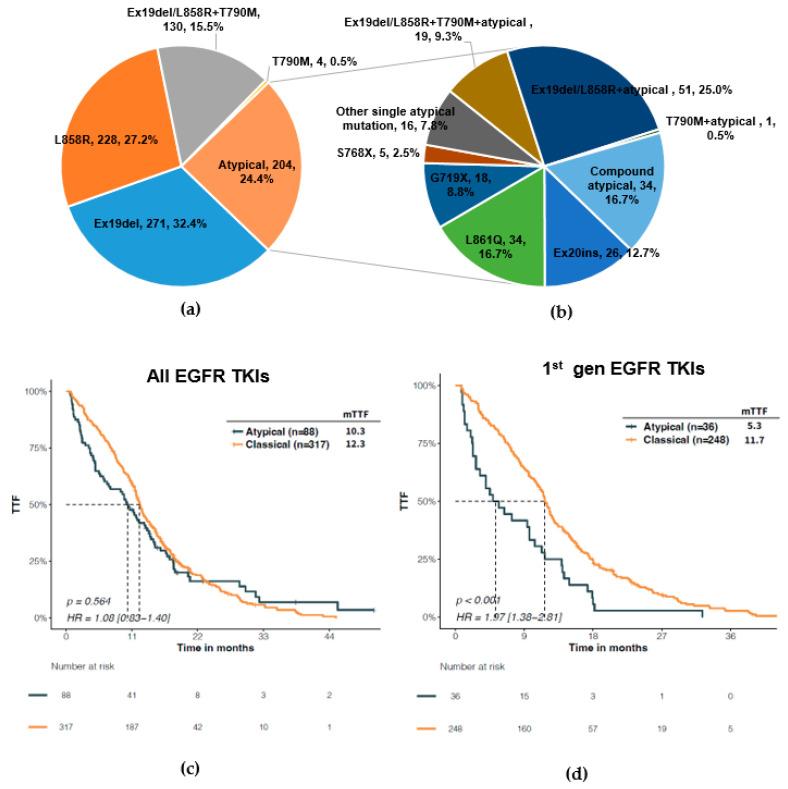

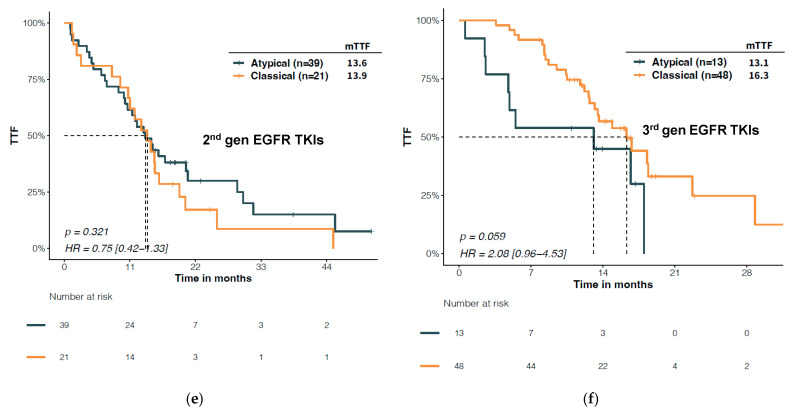
Predictive significance of classical and atypical classification of *EGFR* mutations for EGFR-TKI efficacy. (**a**) Distribution of classical and atypical classification of *EGFR* mutations by samples. (**b**) Distribution of atypical *EGFR* mutations in different exons. Comparison of time to treatment failure (TTF) between patients with classical EGFR mutation and those with atypical mutations upon 1st line treatment with all EGFR TKIs (**c**), 1st gen EGFR TKIs (**d**), 2nd gen EGFR TKIs (**e**), or 3rd gen EGFR TKIs (**f**).

Among the 837 *EGFR*-mutant tumor samples, 633 (75.6%) harbored classical *EGFR* mutations, including Ex19del (32.4%, n = 271), L858R (27.2%, n = 228), Ex19del/L858R concomitant with T790M (15.5%, n = 130), and T790M alone (0.5%, n = 4) (Figure 2a). Two hundred and four (24.4%) samples carried atypical *EGFR* mutations. The most common single atypical mutations were Ex20ins (12.7%, n = 26), L861Q (16.7%, n = 34), G719X (8.8%, n = 18), and S768X (2.5%, n = 5). Fifty-one (25.0%) samples harbored an atypical mutation concomitant with classical 19del or L858R and 20 (9.8%) had an atypical mutation concurring with T790M (with or without 19del/L858R). Thirty-four samples had compound mutations of only atypical kinds (16.7%) (Figure 2b). The proportion of atypical mutations was similar between the TKI treatment naïve (24.9%) and TKI resistant patients (21.7%). Most atypical mutations in the TKI resistant samples co-occurred with a classical mutation 19del/L858R with (21.1%) or without T790M (33.3%), while this proportion was 19.6% in the TKI treatment naïve samples. Ex20ins (21.5% vs. 1.8%) and G719X (13.1% vs. 3.5%) comprised higher proportions of atypical mutations in TKI naïve vs. resistant samples (Figure S1).

Patients with an atypical *EGFR* mutation had comparable TTF vs. those with classical mutations upon EGFR TKI as 1st line treatment (Figure 2c). This result is against previous knowledge that atypical *EGFR* mutations have an attenuated responsiveness to EGFR TKIs, suggesting a heterogeneous efficacy of EGFR TKI in this subgroup of patients. Specifically, the heterogeneity was predominantly seen with second generation EGFR TKI: as first line treatment, both first and third generation EGFR TKIs demonstrated a longer TTF in patients with classical mutations versus those with atypical ones, while second generation EGFR TKI showed comparable TTF between two subgroups (Figure 2d–f). Therefore, it is necessary to find a better classification scheme to select patients from this subgroup who may benefit the most from EGFR TKI treatment, most probably second generation.

### 3.3. Structural Classification of EGFR Mutations in the Chinese Cohort

**Figure 3:**
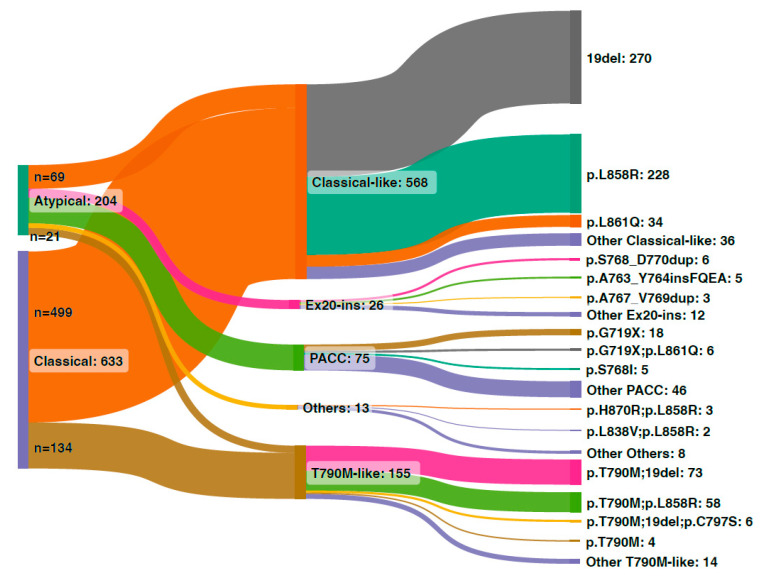
Sankey diagram illustrating the distribution and spectrum of the structural classification of *EGFR* mutations in the Chinese cohort. The left two nodes represent the numbers of classical and atypical mutations. The middle nodes are the structural classification subgroups. The right nodes represent the individual mutations.

According to the structural classification, 568 out of the 837 *EGFR*-mutant samples (67.9%) harbored classical-like mutations, and 155 (18.5%) had the T790M-like type. PACC and Ex20ins-L mutations were identified in 75 (9.0%) and 26 (3.1%) samples. Thirteen (1.6%) samples harbored atypical mutations that failed to fall into any of the four types (Figure 3). Specifically, 33.8% (n = 69) of the atypical mutations were classified into the classical-like group and 36.7% (n = 75) were PACC type. Ex20ins-L and T790M-like types comprised 12.7% and 10.3% of atypical mutations, respectively. Among the PACC mutations, p. G719X (24.0%, n = 18), concurrent p. G719X and p. L861Q (8.0%, n = 6), and p.S768I (6.7%, n = 5) were the most prevalent. Compound mutations including p. G719X and p. S768I accounted for 6.7% (n = 5). Twelve (16%) were compound mutations with p. G719X and other mutations. p.S768_D770dup (23.1%, n = 6) and p.A763_Y764insFQEA (19.2%, n = 5) were the most common Ex20ins-L mutations. The most common classical-like atypical mutation was p. L861Q (6.0%). Ex19del concomitant with T790M-C797S was the major T790M-like atypical mutation (3.9%).

### 3.4. Predictive Significance of Structural Classification of EGFR Mutations

**Figure 4:**
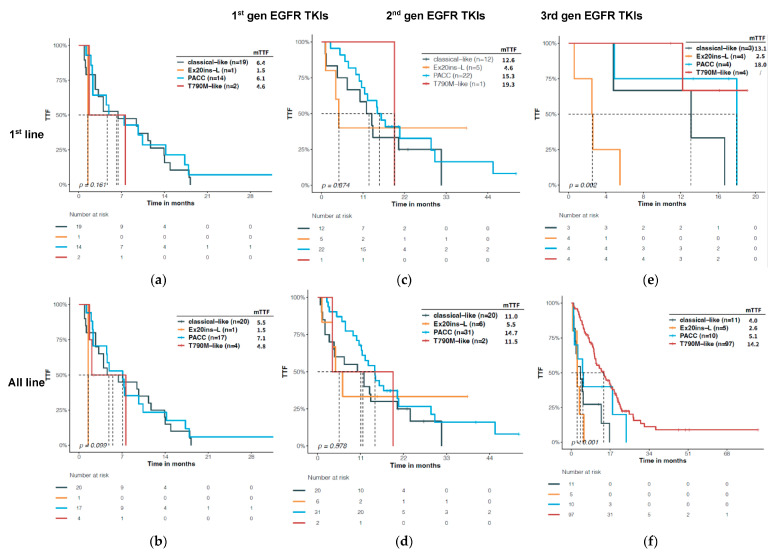
Predictive significance of the structural classification on *EGFR* mutations for different EGFR TKIs. Time to treatment failure (TTF) was compared among different structural groups upon 1st, 2nd, and 3rd gen EGFR TKI as 1st line treatment (**a**–**c**) or regardless of treatment line (**d**–**f**). 19del and L858R were excluded from the classical-like subset for this analysis.

Next, we interrogated the predictive value of the structural classification on the efficacy of various EGFR TKIs. The TTF upon first generation TKI was comparable between patients with the classical-like atypical mutations (first line: 6.4 months, all lines: 5.5 months) and those with the PACC mutations (first line: 6.1 months, all lines: 7.1 months) (Figure 4a,b). Upon treatment with second generation TKI, patients with PACC mutations revealed a first line median TTF of 15.3 months and a median TTF of 14.7 months, regardless of line. Comparatively, patients with classical-like atypical mutations had numerically shorter mTTF (first line: 12.6 months; all lines: 11.0 months), although the difference was not significant (*p* = 0.371, *p* = 0.186) (Figure 4c,d). The sample sizes for Ex20ins-L and T790M-like mutations were limited for the first and second gen EGFR TKIs. In response to third generation EGFR TKI, patients with Ex20ins-L mutations showed the shortest first line mTTF (2.5 months, *p* = 0.002) (Figure 4e). T790M-like mutations resulted in the longest TTF (14.2 months, *p* < 0.001) (Figure 4f), which was mainly driven by the 19del/L858R + T790M (Figure S1B).

**Figure 5:**
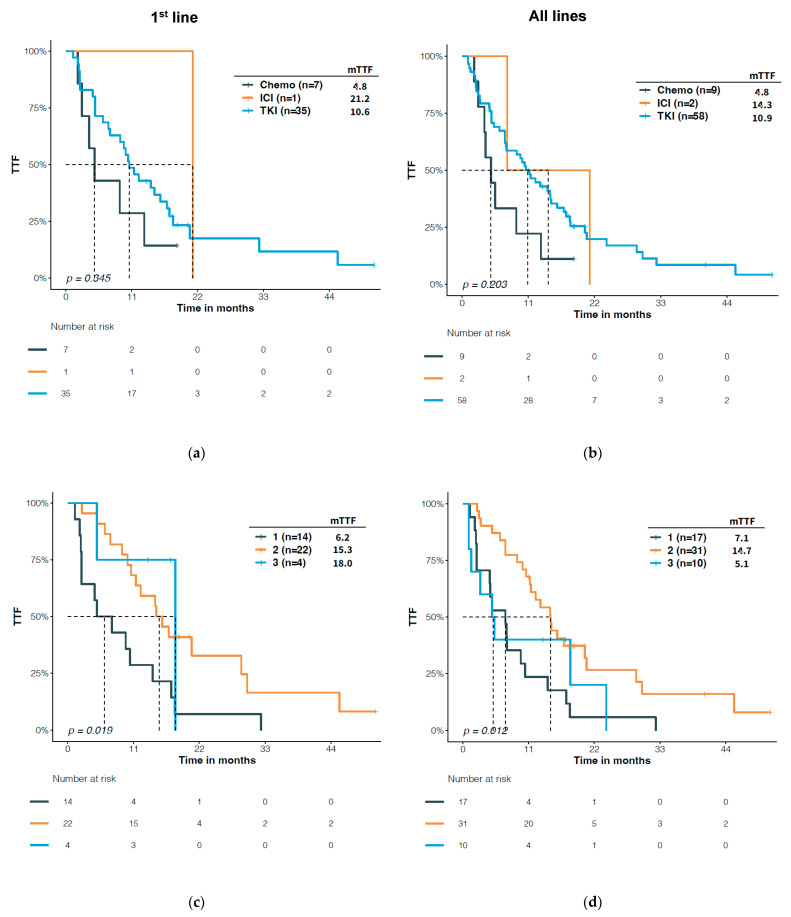

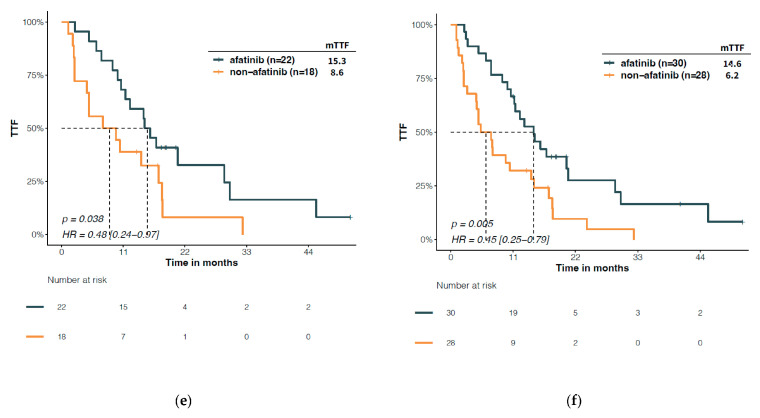
Differential time to treatment failure (TTF) in patients with PACC *EGFR* mutations with different treatments. (**a**,**b**) Comparison among the EGFR TKI, chemotherapy, and immune checkpoint inhibitor (ICI). (**c**,**d**) Comparison among 1st, 2nd, and 3rd gen EGFR TKIs. (**e**,**f**) Comparison between afatinib and other EGFR TKIs. Non-afatinib treatment includes osimertinib (n = 4), erlotinib (n = 1), gefitinib (n = 4), and icotinib (n = 9) for 1st line, and osimertinib (n = 9), erlotinib (n = 3), gefitinib (n = 5), icotinib (n = 9), ametinib (n = 1), and dacomitinib (n = 1) for all lines.

We also compared the TTF with certain structural types of *EGFR* mutations among patients treated with different regimens. TKI treatment tended to result in a longer TTF in patients with PACC mutations compared with chemotherapy (10.9 vs. 4.8 months, *p* = 0.085, Figure 5b). Among the EGFR TKIs, the second generation TKI demonstrated a significantly longer TTF than the first generation TKI (first-line: 15.3 vs. 6.2 months, *p* = 0.009; all-line: 14.7 vs. 7.1 months, *p* = 0.003), and a trend of longer TTF than the third generation TKI (all-line: 14.7 vs. 5.1, *p* = 0.135) (Figure 5c,d). Notably, most patients treated with second generation TKI received afatinib. Patients with PACC mutations had more prolonged TTF when treated with afatinib than when treated with other EGFR TKI either as first line treatment (15.3 vs. 8.6 months, HR = 0.48, *p* = 0.038, Figure 5e) or regardless of treatment line (14.6 vs. 6.2, HR = 0.45, *p* = 0.005, Figure 5f).

**Figure 6:**
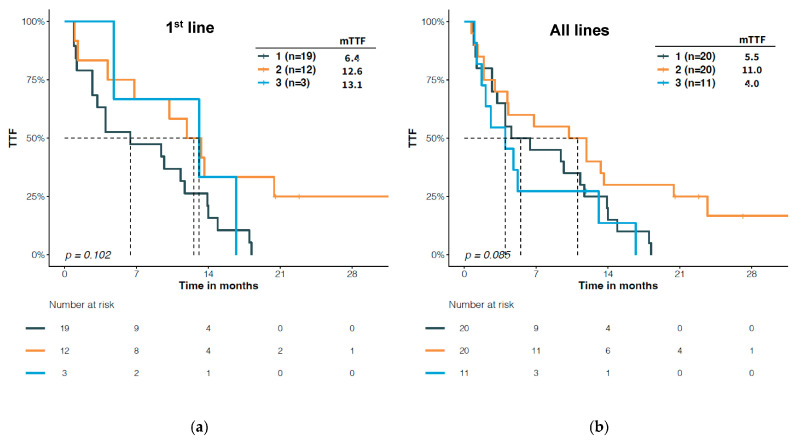
Differential time to treatment failure (TTF) in patients with atypical, classical-like *EGFR* mutations upon different generation EGFR TKIs. (**a**) 1st line treatment. (**b**) All lines.

Interestingly, patients who harbored classical-like atypical *EGFR* mutations had a trend of longer TTF when treated with second-generation TKIs than with first-generation TKIs either as first line (12.6 vs. 6.4, months, *p* = 0.042) (Figure 6a) or regardless of treatment line (11.1 vs. 5.5, *p* = 0.065) (Figure 6b). Furthermore, second-generation TKIs also tended to yield a longer TTF than third-generation TKIs in patients with classical-like atypical mutations (all lines: 11.1 vs. 4.0, *p* = 0.092) (Figure 6b).

Despite the small sample size, patients with Ex20ins-L mutations showed comparable TTF upon chemotherapy, ICI, and EGFR TKI treatments (Figure S2A,B). The second-generation EGFR TKIs tended to yield a numerically longer TTF than the other TKIs (5.5 months vs. 2.6 months and 1.5 months, *p* = 0.07, Figure S2D); however, the sample size was too limited to draw any conclusions.

On the other hand, patients with T790M-like mutations had more favorable TTF upon treatment with EGFR TKIs than with chemotherapy (HR = 2.53, *p* = 0.017, Figure S3A). Specifically, the third-generation EGFR TKIs conferred a longer TTF than the other EGFR TKIs in patients with mutations of this structural type (*p* < 0.001, Figure S3B). Notably, the long TTF is most likely driven by the resistant Ex19del/L858R + T790M. In the subset of patients with atypical, T790M-like *EGFR* mutations, chemotherapy yielded a mTTF of 13.5 months and TKI resulted in a mTTF of 5.3 months (*p* = 0.753, Figure S3C). The number of patients was too small for further stratification analysis on different TKIs.

## 4. Discussion

Despite each having a low frequency, atypical *EGFR* mutations as a whole comprise a substantial proportion of *EGFR*-positive cases in NSCLC. Heterogeneous responses of these atypical mutations to EGFR TKIs make it a challenge and unmet need to group these mutations according to sensitivity to different EGFR TKIs, which may facilitate the design of clinical trials and match patients with the optimal regimen. Robichaux et al. recently proposed a structure-based classification on *EGFR* mutations, which predicted the efficacy of different EGFR TKIs better than the conventional exon-based classification [11]. Herein, we investigated the structural classification in a Chinese real-world cohort of patients with advanced *EGFR*-mutant NSCLC to validate its predictive significance. In our cohort, the PACC and classical-like types constitute the majority of atypical mutations (36.7% and 33.8%, respectively). Ex20ins-L and T790M-like accounted for 12.7% and 10.3%, respectively. This distribution is similar to that reported by Robichaux et al. (49% for PACC, 36% for classical-like, 12% for Ex20ins-L, and 3% for T790M-like) [11]. Among all *EGFR*-mutant cases, we observed a proportion of 67.9% for the classical-like type, 18.5% for T790M-like, 9.0% for PACC, and 3.1% for Ex20ins-L mutations. In another study investigating resectable NSCLC, 86.07%, 7.11%, 5.04%, and 1.78% of the *EGFR* mutations were categorized into classical-like, PACC, Ex20ins-L, and T790M-like groups, respectively [13]. A substantial proportion of EGFR TKI-pretreated patients in our cohort conferred a higher proportion of T790M-like mutations in our study compared with the treatment-naïve, resectable setting.

Robichaux and colleagues developed a preclinical model by transfecting mouse BA/F3 cells with different mutant human *EGFR* cDNAs, for which they stratified *EGFR* mutations into different structural groups according to the distinct patterns of sensitivity to EGFR TKI. Among the four structural groups, PACC mutations were more sensitive to second generation TKIs than any other TKIs [11]. They also reported clinical evidence that patients with PACC mutations had a more favorable TTF than those with other types of *EGFR* mutations upon second generation TKI and these patients benefited the most from second generation TKI among all EGFR TKIs. In line with their results, our study also showed that patients with PACC mutations derive the most benefit from afatinib than other EGFR TKIs (Figure 5c–f). The observed TTF (first-line: 15.3 months) was similar to previously reported TTF to afatinib in EGFR-TKI-naïve patients with G719X (14.7 months) or S768I (15.6 months) [14]. Notably, besides the recurrent p. G719X and p.S768I, other PACC mutations, accounting for almost half of this structural type, have no FDA-approved medication. Our results provide evidence supporting the use of secondgeneration TKI in patients with these uncommon PACC mutations.

In our study, we also observed a trend of longer TTF upon second generation TKI (11.05 months) than first (5.5 months) and third generation TKI (4 months) in patients with classical-like, atypical mutations (Figure 6b, *p* = 0.085). In the subset of patients with first line treatment, the difference between the second and third generation TKI was not seen (12.6 months vs. 13.1 months). This result is inconsistent with the in vitro and in vivo findings by Robichaux et al. that classical-like mutations were sensitive to all EGFR inhibitors, particularly third generation [11]. Unfortunately, they did not perform a side-by-side comparison of TTF among different generations of EGFR TKIs in patients with classical-like, atypical mutations. The discrepancy also underscores the gap between the preclinical model and the clinical setting. In our cohort, L861Q constitutes approximately half of the classical-like, atypical mutations. Studies based on the Ba/F3 model system have demonstrated that L861Q showed comparable sensitivities to both afatinib and osimertinib compared with L858R, but less sensitive to erlotinib [15]. Clinical evidence showed that patients with L861Q had an mPFS ranging from 5 to 9 months when treated with gefitinib/erlotinib [16], while afatinib resulted in an mFPS of 8 to 10 months [8,14], and osimertinib showed an mPFS of 15.2 months [10] in patients with L861Q. In our study, the short TTF of patients with classical-like, atypical mutations upon third generation EGFR TKI could be attributable to non-L861Q mutations and may also be explained by the fact that the majority of them were EGFR-TKI pretreated. Nevertheless, further well-designed studies are warranted to investigate the optimal therapeutic options for patients harboring classical-like, atypical mutations.

Using the preclinical model, Robichaux et al. also showed that 20 exon insertions showed heterogeneous sensitivity toward EGFR TKIs, and those classified into the Exon20ins-L structural type only show sensitivity to second generation TKIs. However, they also noted some degree of heterogeneity in drug sensitivity even within Ex20ins-L mutations [11]. In our study, patients with Exon20ins-L mutations showed a trend of longer TTF towards second than first and third generation EGFR TKIs (5.6 months vs. 1.5 months vs. 2.6 months, *p* = 0.071). In a recent large-scale retrospective study, patients with 20 exon insertions showed a comparable short TTF (3.3 months) upon afatinib vs. other EGFR TKI (3.7 months for osimertinib, 1.0 months for gefitinib, *p* = 0.81) [17]. It should be noted the Ex20 insertions analyzed in these two studies were different by definition. These results suggest that the structural-based classification outperforms the conventional definition of *EGFR* mutations in grouping patients according to the efficacy of EGFR TKI. Nevertheless, the TTF of patients with 20Ex20ins-L mutations was poor upon EGFR-TKIs compared with other structural groups, regardless of TKI generation (Figure 4). Chemotherapy and ICI also resulted in short TTF (<4 months) (Figure S2A,B). In comparison, mobocertinib and amivantamab, two recently approved drugs targeting *EGFR* Exon 20 insertions, demonstrated an mPFS of 7.3 months and 8.3 months, respectively [18,19], and novel exon 20 insertion-specific drugs are increasingly studied under clinical trials. Given the heterogeneity of exon 20 insertions, identifying which subtype may respond best to specific drugs merits further investigation to tailor the treatment choice to patients.

Notably, we also found 1.6% of the *EGFR* mutations in our cohort that were absent in the study of Robichaux et al. and thus unable to be classified into any of the four structural groups. Patients with some of these unclassified mutations also revealed favorable TTF on EGFR TKIs, driving us to further explore the classification of these mutations. The sensitivity of these mutations to different EGFR TKIs is under investigation using cell lines transfected with mutant *EGFR* cDNAs.

In conclusion, our study depicted the landscape of structural types of *EGFR* mutations in Chinese NSCLC patients. Our results also suggested that structural classification can serve as a predictive marker for the efficacy of various EGFR TKIs, which would guide therapeutic decision-making.

## Figures and Tables

**Table 1:** Characteristics of patients for the survival analyses.

	Overall (n = 436)
**Sex**	
Female	234 (53.7%)
Male	202 (46.3%)
**Smoking history**	
No	343 (78.7%)
Yes	93 (21.3%)
**Histology**	
ADC	414 (94.9%)
ADSQ	4 (0.9%)
NOS	9 (2.1%)
SCC	9 (2.1%)
**Stage**	
IIIB	6 (1.4%)
IIIC	9 (2.0%)
IVA	108 (24.8%)
IVB	32 (7.3%)
IVC	281 (64.4%)
**Age**	
Median [IQR]	61.0 [53.8, 67.0]
**Therapy**	
**1st gen EGFR TKI**	302
1st-line ^a^	273 (90.4%)
Later-line	29 (9.6%)
**2nd gen EGFR TKI**	124
1st-line ^a^	54 (43.5%)
Later-line	70 (56.5%)
**3rd gen EGFR TKI**	241
1st-line ^a^	56 (23.2%)
Later-line	185 (76.8%)
**Chemotherapy**	93
1st-line ^b^	31 (33.3%)
Later-line	62 (66.7%)
**ICI**	36
1st-line ^b^	8 (22.2%)
Later-line	28 (77.8%)
**Other**	81
1st-line ^b^	14 (17.3%)
Later-line	67 (82.7%)

ADC: adenocarcinoma carcinoma; ADSQ: adenosquamous carcinoma; NOS: not otherwise specified; SCC: squamous cell carcinoma; ICI: immune checkpoint inhibitor. 1st gen EGFR TKIs include gefitinib, erlotinib, and icotinib; 2nd gen EGFR TKIs include afatinib and dacomitinib; 3rd gen EGFR TKIs include osimertinib, ametinib, furmonertinib, zorifertinib, and D-0316. (a) 1st line treatment with EGFR TKI refers to those administered in patients without previous TKI treatment. (b) 1st line treatment with other therapies refers to treatment in treatment naïve patients.

## Data Availability

The data presented in this study are available on request from the corresponding author. The data are not publicly available due to ethical restriction.

## Supplementary Materials

The following supporting information can be downloaded at: https://www.mdpi.com/article/10.3390/jcm12010236/s1. Figure S1: Distribution of classical and atypical EGFR mutations in TKI treatment naïve patients (A) and those developing PD to TKI (B). Figure S2: Comparison of TTF in patients with Ex20ins-L *EGFR* mutation upon different therapies. Figure S3: Comparison of TTF in patients with T790M-like *EGFR* mutation upon different therapies. Table S1: List of mutations covered by ARMS-PCR. Table S2: List of *EGFR* mutations by structural classification.
