# Supplementary material for: Landscape and Predictive Significance of the Structural Classification of EGFR Mutations in Chinese NSCLCs: A Real-World Study
Source: J Clin Med. 2022 Dec 28;12(1):236. doi: 10.3390/jcm12010236 (PMC9821726; doi:10.3390/jcm12010236)
Supplement: Supplementary file 1 [file jcm-12-00236-s001.zip › Supplementary figures - R2.pptx]

## Slide 1
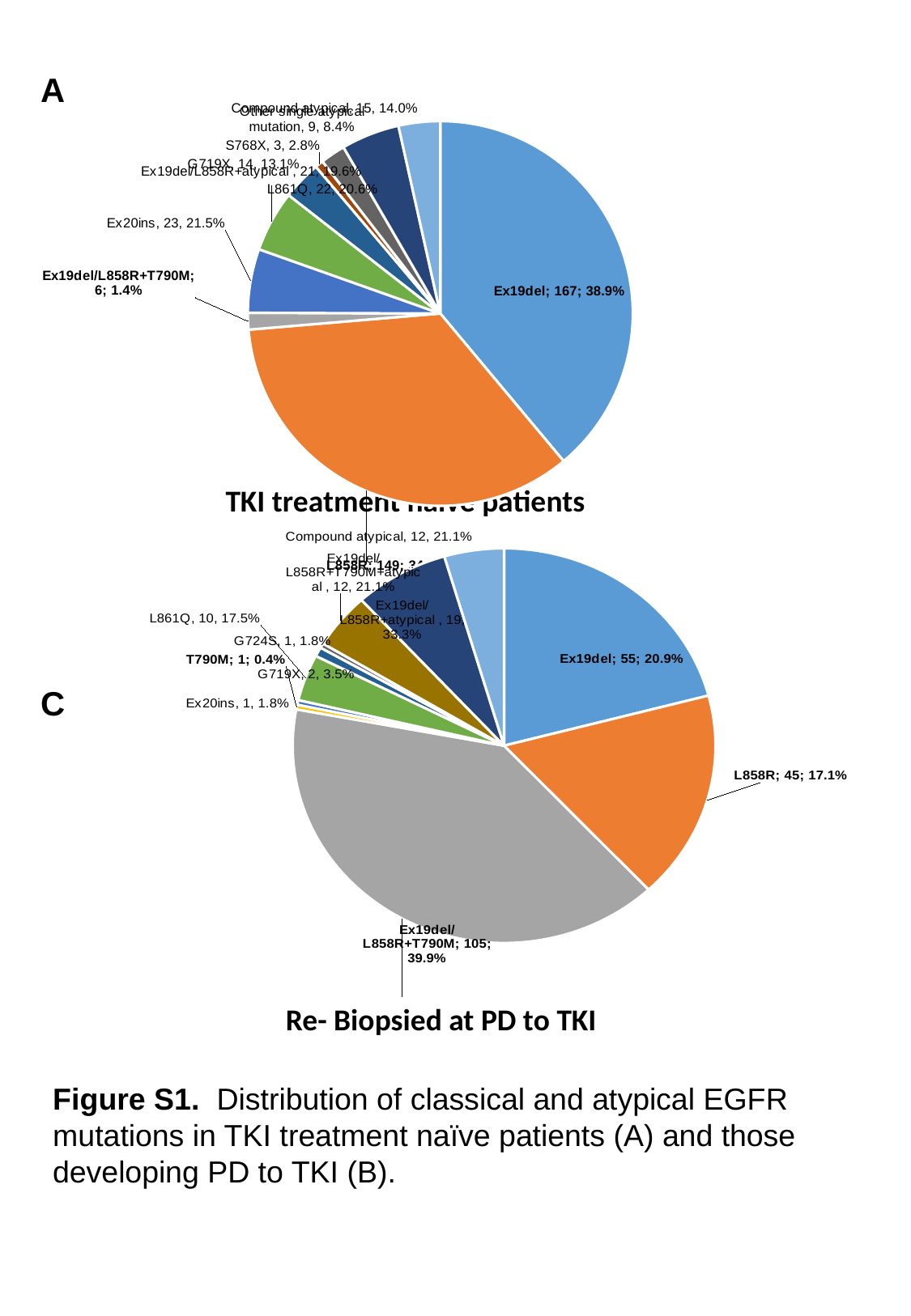

A
[unsupported chart]
TKI treatment naïve patients
[unsupported chart]
[unsupported chart]
[unsupported chart]
C
Re- Biopsied at PD to TKI
Figure S1. Distribution of classical and atypical EGFR mutations in TKI treatment naïve patients (A) and those developing PD to TKI (B).

## Slide 2
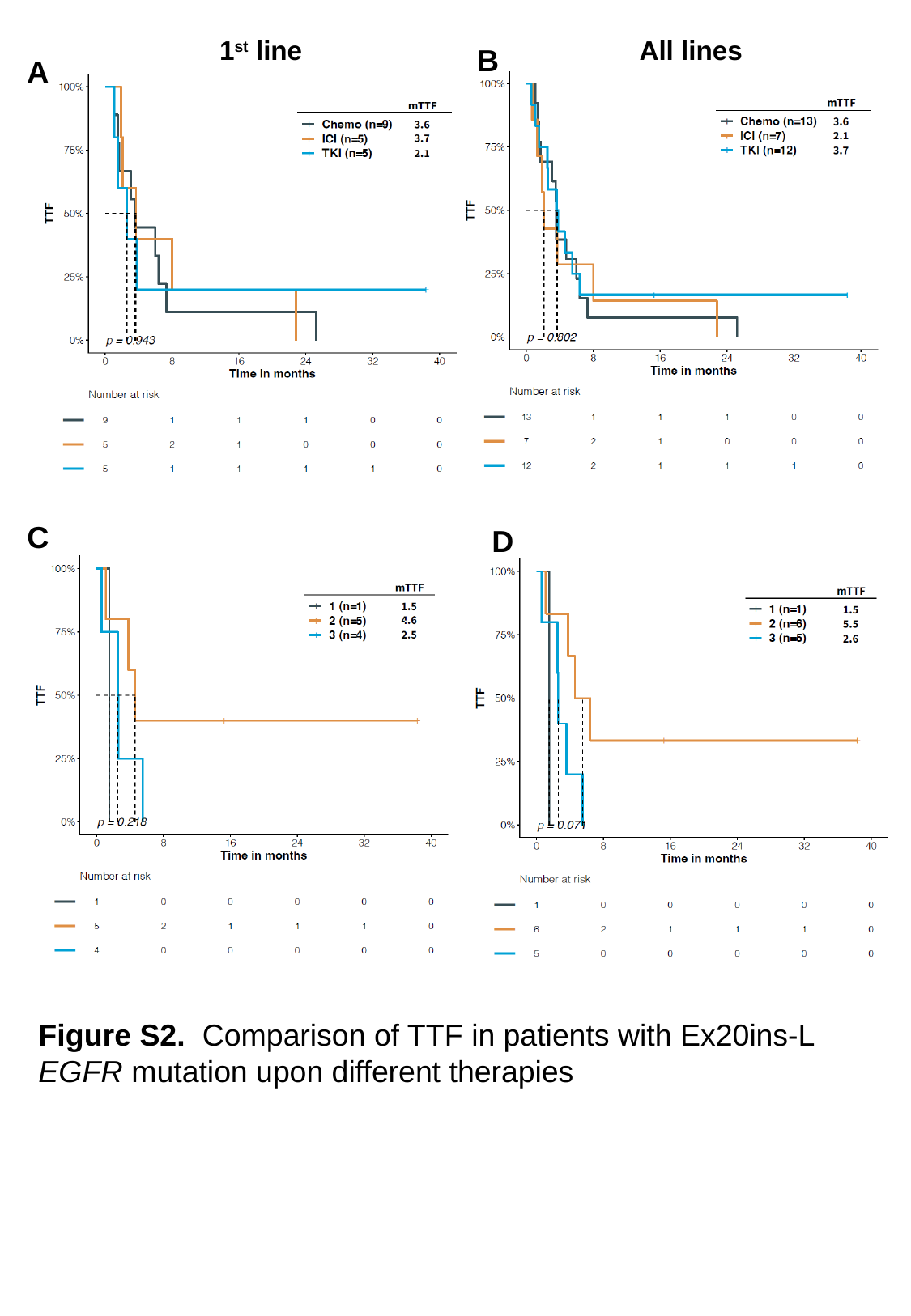

1st line
All lines
B
A
C
D
Figure S2. Comparison of TTF in patients with Ex20ins-L EGFR mutation upon different therapies

## Slide 3
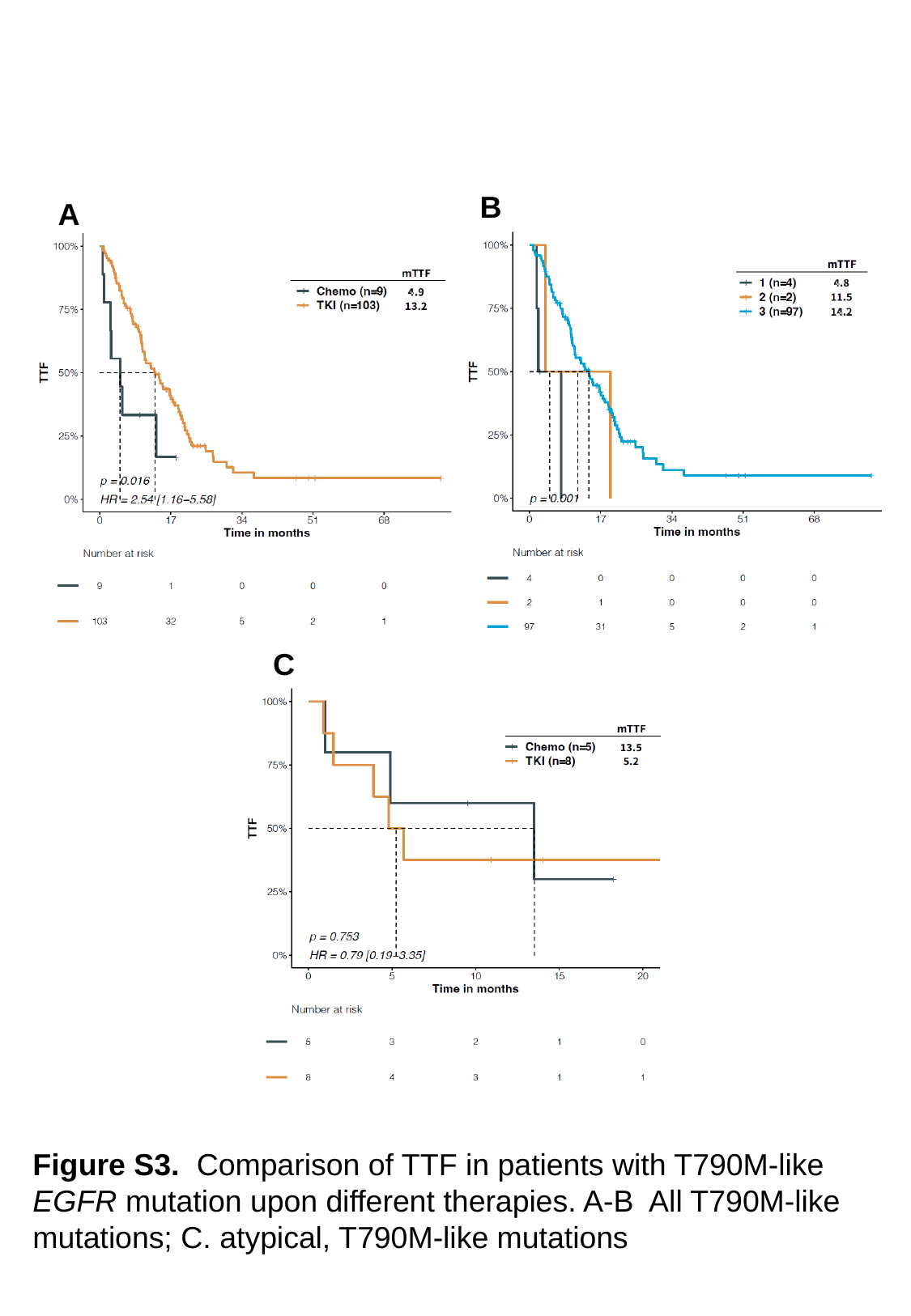

B
A
C
Figure S3. Comparison of TTF in patients with T790M-like EGFR mutation upon different therapies. A-B All T790M-like mutations; C. atypical, T790M-like mutations
